# Characteristics and correlates of sleep duration, daytime napping, snoring and insomnia symptoms among 0.5 million Chinese men and women

**DOI:** 10.1016/j.sleep.2017.11.1131

**Published:** 2018-04

**Authors:** Yiping Chen, Christiana Kartsonaki, Robert Clarke, Yu Guo, Canqing Yu, Zheng Bian, Qilian Jiang, Shanpeng Li, Junshi Chen, Liming Li, Zhengming Chen, China Kadoorie Biobank Study (members listed at end of report)

**Affiliations:** a Medical Research Council Population Health Research Unit, Nuffield Department of Population Health, University of Oxford, Oxford, UK; b Clinical Trial Service Unit & Epidemiological Studies Unit (CTSU), Nuffield Department of Population Health, University of Oxford, Oxford, UK; c Chinese Academy of Medical Sciences, Dong Cheng District, Beijing 100730, China; d Department of Epidemiology and Biostatistics, School of Public Health, Peking University Health Science Center, Beijing, China; e NCDs Prevention and Control Department, Liuzhou CDC, Liuzhou 545007, China; f Qingdao CDC, 175 Shandong Road, Qingdao, Shandong 266033, China; g China National Center for Food Safety Risk Assessment, Chaoyang District, Beijing 100021, China

**Keywords:** Sleep duration, Insomnia symptoms, Mental and physical health

## Abstract

**Background:**

Inadequate sleep duration and insomnia can affect both physical and mental health. There is limited evidence, however, on characteristics and correlates of sleep patterns and insomnia in urban and rural China.

**Methods:**

This cross-sectional study, involving 512,891 adults aged 30–79 years from ten (five urban and five rural) diverse areas in China, recorded detailed information, using interviewer-administered laptop-based questionnaires, on sleep patterns (duration, daytime napping and snoring) and insomnia symptoms. Logistic regression was used to examine the associations of sleep patterns and insomnia symptoms with a range of socio-economic, lifestyle, behaviour and health-related factors.

**Results:**

Overall, the mean (SD) sleep duration was 7.38 (1.37) h, with 23% reporting short (≤6 h) and 16% reporting long (≥9 h) sleep duration, 21% taking daytime naps and 22% having frequent snoring. Overall, 17% reported having insomnia symptoms, with a higher proportion in women than in men (19% vs 13%), in rural than in urban residents (19% vs 15%), and in individuals who were living alone (23%). The adjusted odds ratios (ORs) of having insomnia symptoms were significantly higher among people with major depressive episodes (6.10, 95% CI: 5.69–6.55), generalised anxiety disorders (7.46, 6.65–8.37) and any chronic diseases (1.46; 1.44–1.49). In contrast, the ORs of insomnia symptoms were significantly lower among those reporting napping (0.77, 0.75–0.78) and frequent snoring (0.86, 0.84–0.87).

**Conclusions:**

Among Chinese adults, sleep patterns varied greatly by socio-economic, lifestyle and health-related factors. The risk of insomnia symptoms was associated with both poor mental and physical health status.

## Highlights

• Men, older people, and rural residents in China had longer mean sleep duration than their counter parts.
• Low socio-economic status and living alone were associated with short sleep duration, and low BMI were associated with insomnia symptoms.
• Mental disorders such as depression and anxiety were associated with much greater risk of insomnia symptoms than physical illnesses.

## 1 Introduction

Insomnia is a common problem in middle-aged and older people and has been associated with poor health status. Recent systematic reviews of prospective studies conducted mainly in Western populations have demonstrated that individuals who reported either short sleep (ie <6 h) or long sleep duration (ie >9 h) had a higher prevalence of cardiovascular risk factors (hypertension, diabetes and obesity) and higher risks of both cardiovascular disease and all-cause mortality [1], [2]. However, relatively little is known about the correlates of sleep duration or sleep disturbances or about the characteristics and determinants of sleep duration and insomnia in low- and middle-income countries such as China.

In recent decades in China, there has been a rapid economic transition accompanied by major changes in lifestyle, including changes in sleep duration and sleep habits. The increased use of night-time TV, internet, mobile phones and social media has resulted in reduced sleep duration and increased prevalence of insomnia. Such changes varied substantially by demographic, socio-economic, lifestyle and health factors. Previous studies of the correlates and health consequences of sleep patterns and insomnia in China have been constrained by small sample size [3], collection of only limited data on other lifestyle, behavioural and health-related correlates, and restriction to local occupational or urban cohorts [4]. We report on findings from a nationwide study of 0.5 million Chinese adults in the China Kadoorie Biobank (CKB). The aims of the present study were to examine the following: the patterns of sleep duration, napping, snoring and prevalence of insomnia symptoms, overall and in certain population subgroups (eg by age, gender and region); the socio-economic and lifestyle correlates of sleep duration (short and long) and insomnia symptoms and the association of sleep duration and insomnia symptoms with physical and mental disorders (eg depression and anxiety).

## 2 Methods

### 2.1 Study participants

Details of the design and methods of the CKB study have been described elsewhere [5], [6]. Briefly, 512,891 women and men, aged 30–79 years (mean 52 years), were recruited between 2004 and 2008 from 1175 local communities across ten (five rural and five urban) geographically defined areas in China. The regions were selected according to local disease patterns, exposure to certain risk factors, population stability, qualities of local death and disease registries, local commitment and capacity. In each chosen area, permanent residents aged 30–79 years old were identified through local residential records and invited to participate in the study by letter and information leaflet. Overall, approximately 30% (33% rural, 27% urban areas) of potentially eligible residents participated in the study. Non-participants were mainly those who were absent from home or reluctant to spend time to visit the local assessment centre according to anecdotal reports by field staff. Periodic resurveys of ∼5% of randomly selected surviving participants were conducted using identical approaches. Ethics approval was obtained from all relevant local, national and international authorities prior to commencement of the study. All participants provided written informed consent.

The baseline survey and subsequent re-surveys were conducted at local assessment centres set up specifically for the study. Trained health workers administered laptop-based questionnaire on demographic and socio-economic status, prior medical history, health status, smoking, alcohol consumption, diet, physical activity and other aspects of lifestyle (http://www.ckbiobank.org). The questionnaire had built-in checks to identify and minimise missing items, data entry errors and inconsistencies. Physical measurements including height, weight, waist and hip circumference, heart rate and blood pressure were also recorded. A 10-ml non-fasting blood sample (with time since last meal recorded) was collected for long-term storage. Standard operational procedures, training manuals and onsite training were provided to the local study team in each of ten areas. All devices were regularly maintained and calibrated to ensure consistency of the measurements across the ten areas and over time.

### 2.2 Assessment of insomnia symptoms, napping, snoring and sleep duration

Participants were first asked whether they had any of the following four symptoms in at least three days or more in a week during the last month: (i) taking >30 min to fall asleep after going to bed or waking up in the middle of the night, (ii) waking up early and not being able to go back to sleep, (iii) needing to take medicine (including herbal or sleeping pills) at least once a week to help sleep and (iv) having difficulty staying alert while at work, eating or meeting people during daytime. Participants who answered ‘Yes’ to any of the above i, ii and iii symptoms were classified as having insomnia symptoms (Section 10.3 Baseline questionnaire: http://www.ckbiobank.org/site/binaries/content/assets/resources/pdf/qs_baseline-final-from10june2004.pdf).

Subsequently, participants were asked the following multiple choice questions: Do you usually take a daytime nap? (‘Yes, usually’; ‘Yes, but only in summer’ and ‘No’); Do you snore during sleep? (‘Yes, Frequently’; ‘Yes, Sometimes’ or ‘No/Don't know’). Finally, participants were asked ‘How many hours do you typically sleep per day including naps?’

### 2.3 Assessment of physical and mental health

Participants were asked if they had ever been diagnosed by a doctor as having any major diseases, including ischaemic heart disease, stroke/TIA, emphysema/chronic bronchitis, diabetes mellitus and cancer (Section 7 Baseline questionnaire: http://www.ckbiobank.org).

Participants were assessed using the CIDI-SF (A) questionnaire for major depressive episode (MDE) and CIDI-SF (B) for generalised anxiety disorder (GAD) [7]. Those who met the CIDI-SF criteria (A) were classified as having had a MDE, and those who met CIDI-SF (B) [7] were classified as having GAD. Questions on ten major stressful life events related to death of spouse, family conflict, financial difficulty, violence, etc. were also included, along with level of life satisfaction and level of self-rated health status (http://www.ckbiobank.org/site/binaries/content/assets/resources/pdf/qs_baseline-final-from10june2004.pdf).

### 2.4 Statistical methods

The adjusted mean sleep duration (in hours) and proportions of individuals reporting insomnia symptoms, napping and frequent snoring were calculated by direct standardisation to the age (in ten year groups), gender and study area (ten groups) structure of the CKB cohort. Logistic regression was used to estimate adjusted odds ratios (OR) of short (≤6 h) and long (≥9 h) sleep duration (reference group: 7–8 h) and OR of insomnia symptoms by selected baseline characteristics adjusted for age, gender, region, body mass index (BMI) (five groups: <18.5, 18.5–24.99, 25–29.99, 30–34.99 and ≥35 kg/m^2^), systolic blood pressure (SBP mmHg) (<110, 110–129, 130–139, >140), smoking (three groups: never regular, former regular and current regular smoker), alcohol (four groups: never regular, former regular, occasional and weekly drinkers) and metabolic equivalent of task (MET) (four groups: <10, 10–19.99, 20–29.99 and ≥30 MET-h per day). For variables with three or more groups, ‘floating’ standard errors [8] were used to facilitate comparisons between any two groups. These analyses were repeated after excluding individuals with prior disease (any of ten diseases reported at study entry) and MDE or GAD as sensitivity analyses. All analyses were conducted in R version 3.3.1 (R Core Team, 2016).

## 3 Results

**Fig. 1:**
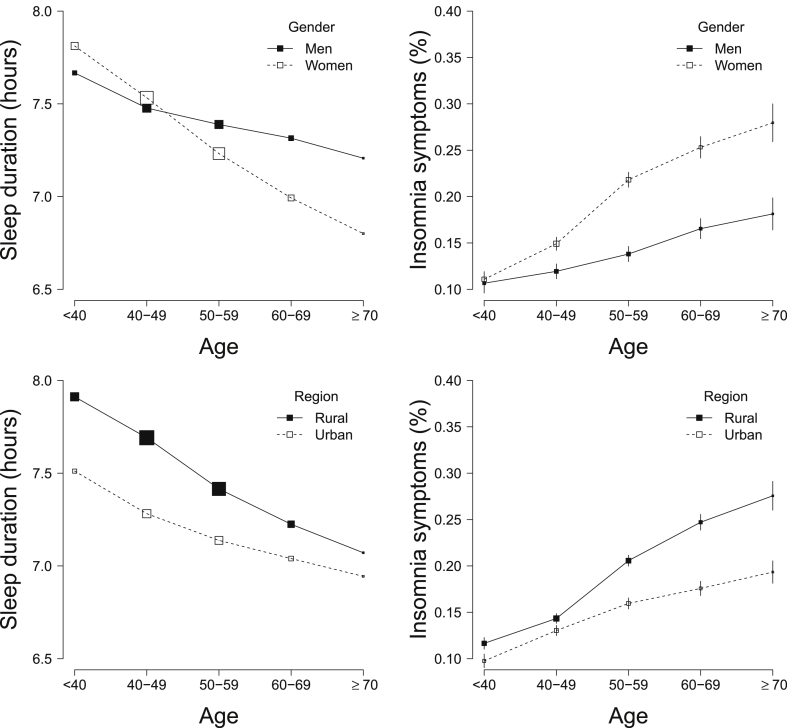
**Mean sleep duration and proportion of insomnia symptoms by age, gender and region**. Solid squares represent men or rural residents; open squares represent women or urban residents.

The overall mean (SD) sleep duration was 7.38 (1.37) h and was higher (7.46 h) among those without prior diseases, MDE or GAD (Table 1 and Web Table 1). The duration of sleep decreased with age, whereas the converse was true for the proportion of individuals reporting insomnia symptoms (Fig. 1). Compared with men, women had slightly shorter sleep duration (7.35 vs 7.43 h; Table 1) and a higher proportion of insomnia symptoms, especially after age 50 years. Participants living in urban areas had shorter sleep duration (7.21 vs 7.51 h; Table 1), but a lower proportion reported insomnia symptoms across all age groups (Fig. 1). The patterns were unaltered after excluding individuals with prior diseases, MDE or GAD (Web Table 1). The proportion of individuals reporting daytime napping was higher in men than in women, in urban than in rural areas (36.4% vs 8.5%) and increased with age (Table 1). However, the proportion reporting only seasonal napping (ie napping only in the summer) was much higher in rural than in urban areas (58.3% vs 16.3%; data not shown). The proportion reporting frequent snoring was higher in men, in urban residents and those with annual household income more than 30,000 RMB (Table 1).

**Table 1:** Mean sleep duration and proportion of individuals reporting insomnia symptoms according to selected characteristics of participants.

	N	Mean sleep duration (h)	Insomnia symptoms (%)	Daytime naps (%)	Frequent snoring (%)	Short sleep (≤6 h) (%)	Long sleep (≥9 h) (%)
**All**	512,891	7.38	16.8	20.9	22.0	23.1	15.9
**Age (year)**							
30-39	77,804	7.73	10.8	18.2	13.6	12.9	19.6
40-49	152,748	7.5	13.8	19.4	19.9	19.0	16.1
50-59	157,556	7.29	18.5	20.4	26.4	25.3	14.4
60-69	91,773	7.14	21.7	24.2	26.0	30.8	14.6
70-79	33,010	6.98	24.8	26.5	21.8	37.2	15.0
**Gender**							
Men	210,259	7.43	13.5	24.9	28.9	21.7	15.6
Women	302,632	7.35	19.2	18.0	17.5	24.2	16.0
**Region**							
Rural	286,705	7.51	18.7	8.5	20.4	21.2	20.3
Urban	226,186	7.21	14.6	36.4	24.1	25.6	10.3
**Highest education**							
No formal school/Primary school	260,437	7.37	17.9	18.8	22.5	24.7	17.1
Middle school/High school	222,440	7.4	15.8	22.9	22.0	22.0	15.4
Technical school/College/University	30,014	7.42	14.0	40.5	21.8	16.7	12.4
**Annual household income (yuan)**							
<10,000	144,832	7.37	20.1	18.1	21.1	25.5	18.4
10,000–19,999	149,013	7.41	16.9	20.0	22.2	23.0	17.1
20,000–34,999	126,721	7.39	15.9	23.5	22.6	22.5	15.1
≥35,000	92,325	7.4	15.5	26.8	23.8	21.4	14.9
**Marital status**							
Married	464,608	7.4	16.4	21.1	22.3	22.6	16.0
Widowed	36,572	7.14	23.7	18.9	19.9	30.7	13.5
Separated/divorced	7945	7.2	20.4	20.6	20.8	28.4	15.1
Never married	3766	6.92	17.6	18.2	14.1	25.2	15.9
**Living alone**							
No	498,335	7.39	16.6	20.9	22.1	22.9	16.0
Yes	14,556	6.92	23.4	19.8	17.7	31.7	13.9
**Occupation**							
Manual worker	286,402	7.31	16.5	16.3	21.6	22.6	15.0
Not in employment	138,460	7.43	17.9	24.9	24.2	23.6	18.4
Office worker	66,173	7.36	14.9	25.7	23.9	21.4	13.5
Other or not stated	8267	7.19	17.2	20.1	24.5	23.1	19.3
Unemployed	13,589	7.13	21.1	22.6	21.8	25.2	16.5
**Smoking status**							
Never regular smoker	346,773	7.38	16.9	21.1	21.0	23.1	15.7
Ex-regular smoker	30,563	7.09	21.0	23.7	28.6	33.5	15.1
Regular smoker	135,555	7.36	20.1	20.7	26.4	24.6	17.5
**Alcohol**							
Never regular drinker	235,199	7.41	16.9	20.8	21.1	23.0	17.0
Ex-regular drinker	9256	6.95	23.2	23.8	23.2	27.5	16.0
Occasional drinker	192,284	7.38	16.8	21.1	22.2	22.8	15.8
Weekly drinker	76,152	7.37	18.3	25.0	24.0	23.7	15.8
**Physical activity (MET**^a^**-h/day)**							
<10	121,598	7.44	18.0	22.3	23.5	24.0	19.6
10-29	262,808	7.39	16.5	21.6	22.1	22.5	15.7
≥30	128,485	7.28	16.5	17.6	21.7	24.5	13.4
**BMI**^b^**(kg/m**^b^**)**							
<18.5	22,373	7.23	22.4	18.6	10.9	27.8	14.8
18.5–24.99	321,586	7.37	17.4	20.3	17.2	23.4	15.7
25–29.99	147,965	7.43	15.0	22.4	31.1	22.0	16.6
≥30	20,965	7.44	13.6	23.4	48.4	22.3	17.5
**Waist Circumference (mm)**							
200–699	71,879	7.30	20.3	18.2	12.5	25.5	15.3
700–799	187,350	7.36	17.5	19.5	16.1	23.7	15.7
800–899	167,260	7.40	15.7	21.5	24.6	22.5	15.9
900–1499	86,402	7.43	14.5	23.3	38.0	22.1	16.9
**SBP**^c^**(mmHg)**							
<110	70,853	7.34	17.9	19.8	16.6	23.9	15.0
(110,120)	92,407	7.36	17.0	20.3	18.7	23.5	15.4
(120,130)	113,683	7.37	16.7	20.5	21.0	23.3	15.5
(130,140)	87,936	7.39	16.5	21.2	23.5	22.8	16.1
≥140	148,012	7.41	16.6	21.5	27.7	22.7	16.8

Adjusted for age, region and sex (where appropriate).

a MET: Metabolic Equivalent Task.

b BMI: Body Mass Index.

c Systolic Blood Pressure.

### 3.1 Patterns and correlates of sleep duration, daytime napping and snoring

Overall, about 23% reported short sleep duration (ie ≤6 h), and 16% reported long sleep duration (ie ≥9 h; Table 1). Exclusion of people with prior diseases and 12-month MDE or GAD significantly decreased the proportion of individuals with short but not long sleep duration (Web Table 1). Among people without prior diseases and MDE or GAD, the proportion of individuals reporting short sleep duration was more common at age >70 years, in women, in those with low socio-economic status (including no formal education, annual household income <10,000 RMB and unemployed) and in those with high physical activity (MET >30 h per day) (Web Table 1). The proportion reporting short sleep duration in participants without prior diseases was higher in those who were widowed (28%), living alone (30%), not employed (21%) and ex-regular smokers (28%) (Web Table 1). The proportion of individuals reporting long sleep duration in participants without prior diseases was higher in those with higher BMI (≥30 kg/m^2^) or higher SBP (≥140 mmHg). Although low BMI was associated with short sleep duration (Table 1), the association became U-shaped after excluding prior diseases including 12-month MDE and GAD (Web Table 1).

**Fig. 2:**
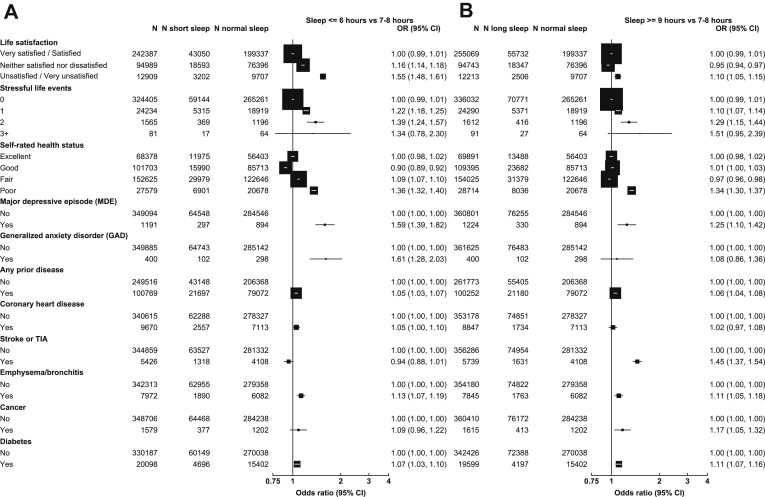
**Adjusted ORs for (A) short sleep duration and (B) long sleep duration by mental health conditions and physical illness, excluding individuals with insomnia symptoms**. Each solid square represents an odds ratio (OR), and the size of the squares is inversely proportional to the variance of the log OR in that group after considering the variance of the log risk in the reference group where appropriate. The horizontal lines represent the 95% CI. All ORs were stratified by age, gender and region and adjusted for BMI, smoking, alcohol, MET-h/day and systolic blood pressure (SBP). Prior disease was defined as having been diagnosed by a doctor with at least one of the following diseases: diabetes, coronary heart disease, stroke or TIA, hypertension, rheumatic heart disease, tuberculosis, emphysema/bronchitis, asthma, cirrhosis/hepatitis, peptic ulcer, gallbladder disease, kidney disease, rheumatoid arthritis, head injury and cancer.

The results for short and long sleep duration after excluding individuals with any insomnia symptoms are shown in Fig. 2A and B, respectively. Compared to people with an average sleep duration of 7–8 h, those reporting short sleep duration (≤6 h) were more likely to have poor life satisfaction (adjusted OR 1.55; 1.48–1.61); two stressful life events (1.39; 1.24–1.57); poor self-rated health status (1.36; 1.32–1.40); 12-month MDE (1.59; 1.39–1.82); 12-month GAD (1.61; 1.28–2.03); and certain major physical illnesses such as emphysema/bronchitis (1.13; 1.07–1.19), coronary heart disease (1.05; 1.00–1.10) and cancer (1.09; 0.96–1.22) (Fig. 2A). Compared to short sleep duration, those reporting long sleep duration (≥9 h) were also more likely to have poor life satisfaction (OR 1.10; 1.05–1.15), two stressful life events (1.29; 1.15–1.44), poor self-rated health status (1.34; 1.30–1.37), 12-month MDE (1.25; 1.10–1.42), 12-month GAD (1.08; 0.86–1.36) and emphysema/bronchitis (1.11; 1.05–1.18). Compared to short sleep duration, individuals reporting long sleep duration were associated with higher risks of stroke (OR 1.45; 1.37–1.54), cancer (1.17; 1.05–1.32) and diabetes (1.11; 1.07–1.16) (Fig. 2B).

Overall, one-fifth of participants reported daytime napping, with a higher proportion in individuals with higher socio-economic status (eg university education, household income ≥35,000 RMB and office worker), married, not living alone, non-smokers and regular drinkers. Moreover, daytime napping was also associated with higher levels of BMI and lower levels of physical activity (Table 1). In the study, almost a quarter of the participants reported frequent snoring, and it was associated with various socio-economic and lifestyle factors similar to those associated with daytime napping. Individuals reporting daytime napping or frequent snoring were less likely to report insomnia symptoms. Snoring was associated with slightly longer sleep duration (5 min; P < 0.0001, data not shown).

### 3.2 Characteristics and correlates of insomnia symptoms

Overall, 16.8% of the participants reported having insomnia symptoms (Table 1). Of those surveyed, 69% had difficulty falling sleep and 64% waking up early. About 7% of individuals with insomnia symptoms used medication, and 14% participants had difficulty staying alert during daytime (Table 2). Among those that answered insomnia questions, 58% had one insomnia symptom, 31% had two insomnia symptoms, and very few had three or more insomnia symptoms (Table 2).

**Table 2:** Proportion of responses to each question among participants with insomnia symptoms. A: > 30 min to fall asleep or waking up in the middle of the night; B: Waking up too early and not being able to go back to sleep; C: Needing medication to sleep (at least once a week); D: Difficulty staying alert during daytime.

	N	Type of symptoms				Number of symptoms			
		A (%)	B (%)	C (%)	D (%)	1 (%)	2 (%)	3 (%)	4 (%)
**All**	86,119	69.3	63.7	7.5	13.7	58.1	31.4	8.7	1.8
**Gender**									
Men	28,867	67.1	62.1	5.9	10.8	63.4	28.7	6.7	1.3
Women	57,252	70.5	64.5	8.3	15.2	55.4	32.9	9.7	2.1

Adjusted for age, sex and region (where appropriate).

Individuals reporting insomnia symptoms were more likely to have lower socio-economic status, with a particularly high rate among those with poor education, lower household income, widowed, living alone, unemployed, ex-regular smokers or ex-regular drinkers (Table 1). Additionally, in all individuals, after adjusting for age, sex, region, smoking, alcohol, MET, snoring, SBP and BMI, hypertension was associated with slightly but statistically significantly longer sleep duration (2 min; P < 0.0001; data not shown), BMI (kg/m^2^) was also significantly associated with longer sleep duration (about 8–9 min in individuals with BMI ≥18.5 compared to underweight individuals; P < 0.0001; data not shown). With the same adjustments, hypertension was not associated with insomnia symptoms, and BMI (kg/m^2^) had an inverse dose-response association with insomnia symptoms (ORs 0.74, 0.62 and 0.56 for BMI 18.5–24.99, 25–29.99 and ≥30, respectively, compared to <18.5; data not shown). These associations remained largely unaltered after excluding prior diseases and 12-month MDE and GAD (Web Table 1).

**Fig. 3:**
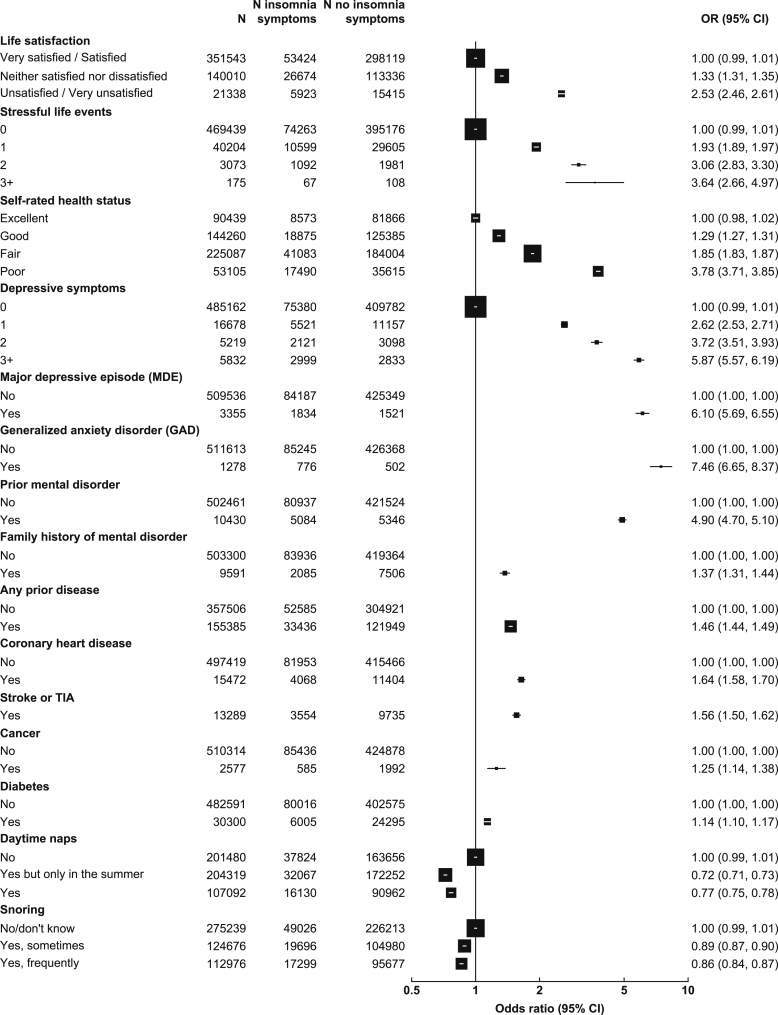
Adjusted ORs for insomnia symptoms by mental health conditions and physical illness, napping and snoring. Symbols and conventions are as in Fig. 2.

Participants who rated themselves unsatisfied or very unsatisfied with their life were much more likely to report insomnia symptoms than those who rated themselves very satisfied (OR: 2.53; 95% CI: 2.46–2.61; Fig. 3). Similarly, participants who rated their health status as poor were also more likely to have insomnia symptoms than those with excellent self-rated health status (OR 3.78; 3.71–3.85; Fig. 3). As with life satisfaction and level of self-rated health, there was a positive dose-response relationship between the number of stressful life events and insomnia symptoms (Fig. 3). Similarly, individuals who had MDE or GAD during the previous 12 months were 6–7 times more likely to have insomnia symptoms, and those with prior history of mental disorders were six times more likely to have insomnia symptoms (Fig. 3). Prior diseases were also found to be associated with moderately increased odds of insomnia symptoms (Fig. 3). Because major life events could affect sleep, in sensitivity analyses, we excluded individuals who experienced major life events, and the results remained unchanged (data not shown). We also examined ORs of insomnia symptoms by physical illness and mental disorders in those with short (≤6 h) and long (≥9 h) sleep duration, but the patterns remained unchanged (data not shown).

## 4 Discussion

This is the largest study to date of sleep patterns and correlates in middle-aged Chinese men and women. The study showed that sleep duration shortened as age increased, and women had shorter sleep duration and more insomnia symptoms at least after 50 years old, whereas rural residents had longer sleep duration but higher proportion of insomnia symptoms. Moreover, short sleep duration and insomnia symptoms were strongly associated with poor mental health status, particularly depression, and, albeit to a lesser extent, with poor physical health status.

Several population-based studies have previously reported associations of either short or long sleep duration among adults with mental and physical diseases. In the present study, the mean sleep duration was 7.46 h among those without major health problems, consistent with those previously reported in much smaller studies in China (7.76 h) conducted during 2009 [3], but appeared to be longer than those reported in other studies in high-income countries such as Korea (7 h) [9] or North America (6.99 h) [10]. In the present study, the total sleep duration also included daytime napping, which is much more common in China than in the US and Korea, which may explain the slightly longer duration observed in the present study.

A few large cross-sectional studies have examined the correlates of sleep duration in high-income countries such as US and Korea [9], [10]. In general, these studies demonstrated that both short (≤6 h) and long (≥9 h) sleep duration were each associated with older age, women and lower SES. The present study in Chinese adults demonstrated similar associations with these factors. The association of short sleep duration with obesity has been reported in both children and adults [11]. A meta-analysis of 17 cross-sectional studies, involving 600,000 individuals aged 15–102 years, showed that short sleep duration was associated with 55% and 89% higher risks of obesity in adults and children, respectively [11]. However, a small cross-sectional study of about 6000 adults aged >15 years in Beijing, China [3], reported that individuals with short sleep duration had lower BMI, similar to what we found in the present study without excluding prior diseases. After excluding those with prior disease and 12-month MDE and GAD, both high BMI and low BMI were associated with short sleep duration. The relationship of BMI with sleep duration in adults warrants further study in prospective studies with longitudinal measurements adjusting for regression dilution of both BMI and sleep duration [12].

Insomnia symptoms were more often associated, although not exclusively, with short sleep duration. We examined the associations of mental and physical illness with short and long sleep duration while excluding participants who reported any insomnia symptoms. Both short and long sleep duration were associated with higher risks of psychiatric disorders including depressive and anxiety disorders in studies conducted in the US [13], Korea [9] and China [3], but most such studies included only a modest number of participants. However, in a Korean study of 6510 adults [9], only short sleep duration was associated with increased risk of depression and anxiety. The present study showed that people with several mental health-related traits (eg poor life satisfaction, poor self-rated health status, stressful life events, and 12-month MDE and GAD) were much more likely to have short and, to a lesser extent, long sleep duration. Short or long sleep duration may represent a marker of susceptibility to psychiatric disorders, including depression (as either may indeed indicate the development of depression) [13]. A meta-analysis of prospective studies, involving 474,684 adults with 16,000 cardiovascular events, showed that both short and long sleep duration were associated with higher risks of cardiovascular diseases [14]. Another US study of over 55,000 adults also showed that in addition to CVD, the risks of hypertension and diabetes were also elevated among people with either short or long sleep duration [2], [15]. The findings of the present study were broadly consistent with previous cross-sectional studies conducted in Chinese adults [4] [16] and with prospective studies [14].

In the present study, insomnia symptoms were comparable to those used in Diagnostic and Statistical Manual of Mental Disorders (DSM-IV-TR) [17] or International Classification of Sleep Disorders (ICSD) [18] and Research Diagnostic Criteria (RDC) for insomnia [19]. Among individuals without apparent physical and mental disorders, the proportion reporting at least one insomnia symptom in the present study (17%) was less than half of that reported in a US population using the same criteria [20] and lower than the estimates in two small cross-sectional studies in China [3], [21], in which sleep disturbance was measured by Pittsburgh Sleep Quality Index (PSQI). The reasons for the apparent discrepancy are not clear but could be due at least in part to the use of a non-representative sample and low overall response rate in the present study, which could lead to underestimation of the prevalence of insomnia symptoms. Insomnia symptoms can be either a risk factor or symptom of mental disorders. A few prospective studies, including the US-based HUNT study of 24,715 people, reported that insomnia predicts long-term risk of several major physical and mental diseases [22], [23]. The present cross-sectional study demonstrated strong positive associations of insomnia symptoms with a range of mental conditions including MDE, GAD and prior mental disorders. Consistent with previous reports [20] [24] the current study found that women, those with older age, lower levels of education and household income, and unemployment were more likely to have insomnia symptoms. Those who were divorced/separated, widowed and/or living alone were also more likely to report insomnia symptoms. The higher proportion of insomnia symptoms in rural residents could be largely attributed to the lower socio-economic status. As a result of rapid urbanisation and introduction of one child policy in China, living alone became particularly common among the elderly rural population, highlighting the need for provision of adequate social support in rural communities.

Unlike sleep duration, few previous studies have examined the relationship between BMI and insomnia symptoms. The present study showed that individuals with low BMI were more likely to have insomnia symptoms, both in those with or without prior physical and mental disorders. These results are consistent with a report from CKB demonstrating that the prevalence of MDE was inversely associated with BMI [25]. These results should be interpreted with caution as only 4% of the present study population was obese. The present study also showed an inverse association of snoring with insomnia symptoms. A possible explanation is that frequent snoring was associated with both higher BMI and longer sleep duration, which were inversely associated with reporting insomnia symptoms (Table 1). The longer sleep duration reported by snorers may be due to sleep apnoea associated with snoring, although it was not explicitly examined in the present study. Hence, snorers, who are at increased risk of apnoea, may exhibit less insomnia symptoms. The association between snoring and insomnia symptoms needs to be investigated further in individuals with and without apnoea in future studies.

The chief strength of the present study included the large sample size and diverse areas covered; the use of internationally recognised criteria for insomnia symptoms, major depression and generalised anxiety disorder; and directly measured physical measurements. Moreover, the information collected covered a range of socio-economic factors, health-related behaviour characteristics, and physical and mental health status, which enabled comprehensive assessment of their associations with sleep duration and insomnia symptoms in a single study.

The present study also has several limitations. First, the main objective of the prospective CKB was to investigate genetic and non-genetic determinants of major chronic diseases rather than population prevalence or incidence of such diseases. Hence, the selection of areas was based on the diversity of risk factor profile and disease patterns rather than representativeness. Because of the low response rate, which is typical of large cohort studies such as CKB, the present study may have underestimated the prevalence of insomnia symptoms as individuals with severe insomnia symptoms may have been less likely to participate. Nonetheless, the patterns of socio-economic and health-related correlates with sleep duration and insomnia symptoms and the association of extreme sleep duration and insomnia symptoms with mental and physical health conditions are likely to be generalisable to the general population. Second, the information collected was self-reported, using questionnaire rather than objective measures, so the data may be subject to recall bias, but the effects would likely be non-differential on the observed associations. Third, because of the cross-sectional nature of the study, the direction of the associations reported in the present study cannot be reliably established. Finally, information about sleep hygiene, shift work or other common sleep disorders such as RLS and sleep apnoea was not recorded.

Among Chinese adults, sleep patterns and insomnia symptoms varied greatly by socio-economic, lifestyle and health-related factors. People with more extreme sleep duration (≤6 h or ≥9 h), with or without insomnia symptoms, were more likely to have concurrent mental and physical health problems. Similarly, insomnia symptoms, regardless of sleep duration, were also associated with higher odds of both mental and physical diseases.

## Contributors

YC, LL, ZC had full access to the data. All authors were involved in study design, conduct, long-term follow-up, analysis of data, interpretation or writing the report.

## China Kadoorie Biobank collaborative group

**International Steering Committee:** Junshi Chen, Zhengming Chen (PI), Rory Collins, Liming Li (PI), Richard Peto.

**International Co-ordinating Centre, Oxford:** Daniel Avery, Ruth Boxall, Derrick Bennett, Yumei Chang, Yiping Chen, Zhengming Chen, Robert Clarke, Huaidong Du, Simon Gilbert, Alex Hacker, Mike Hill, Michael Holmes, Andri Iona, Christiana Kartsonaki, Rene Kerosi, Ling Kong, Om Kurmi, Garry Lancaster, Sarah Lewington, Kuang Lin, John McDonnell, Iona Millwood, Qunhua Nie, Jayakrishnan Radhakrishnan, Paul Ryder, Sam Sansome, Dan Schmidt, Paul Sherliker, Rajani Sohoni, Becky Stevens, Iain Turnbull, Robin Walters, Jenny Wang, Lin Wang, Neil Wright, Ling Yang, Xiaoming Yang.

**National Co-ordinating Centre, Beijing:** Zheng Bian, Yu Guo, Xiao Han, Can Hou, Jun Lv, Pei Pei, Chao Liu, Biao Jing, Yunlong Tan, Canqing Yu.

**10 Regional Co-ordinating Centres:**

**Qingdao** Qingdao CDC: Zengchang Pang, Ruqin Gao, Shanpeng Li, Shaojie Wang, Yongmei Liu, Ranran Du, Yajing Zang, Liang Cheng, Xiaocao Tian, Hua Zhang, Yaoming Zhai, Feng Ning, Xiaohui Sun, Feifei Li. Licang CDC: Silu Lv, Junzheng Wang, Wei Hou. **Heilongjiang** Provincial CDC: Mingyuan Zeng, Ge Jiang, Xue Zhou. Nangang CDC: Liqiu Yang, Hui He, Bo Yu, Yanjie Li, Qinai Xu, Quan Kang, Ziyan Guo. **Hainan** Provincial CDC: Dan Wang, Ximin Hu, Hongmei Wang, Jinyan Chen, Yan Fu, Zhenwang Fu, Xiaohuan Wang. Meilan CDC: Min Weng, Zhendong Guo, Shukuan Wu, Yilei Li, Huimei Li, Zhifang Fu. **Jiangsu** Provincial CDC: Ming Wu, Yonglin Zhou, Jinyi Zhou, Ran Tao, Jie Yang, Jian Su. Suzhou CDC: Fang Liu, Jun Zhang, Yihe Hu, Yan Lu, Liangcai Ma, Aiyu Tang, Shuo Zhang, Jianrong Jin, Jingchao Liu. **Guangxi** Provincial CDC: Zhenzhu Tang, Naying Chen, Ying Huang. Liuzhou CDC: Mingqiang Li, Jinhuai Meng, Rong Pan, Qilian Jiang, Jian Lan, Yun Liu, Liuping Wei, Liyuan Zhou, Ningyu Chen, Ping Wang, Fanwen Meng, Yulu Qin, Sisi Wang. **Sichuan** Provincial CDC: Xianping Wu, Ningmei Zhang, Xiaofang Chen, Weiwei Zhou. Pengzhou CDC: Guojin Luo, Jianguo Li, Xiaofang Chen, Xunfu Zhong, Jiaqiu Liu, Qiang Sun. **Gansu** Provincial CDC: Pengfei Ge, Xiaolan Ren, Caixia Dong. Maiji CDC: Hui Zhang, Enke Mao, Xiaoping Wang, Tao Wang, Xi Zhang. **Henan** Provincial CDC: Ding Zhang, Gang Zhou, Shixian Feng, Liang Chang, Lei Fan. Huixian CDC: Yulian Gao, Tianyou He, Huarong Sun, Pan He, Chen Hu, Xukui Zhang, Huifang Wu, Pan He. **Zhejiang** Provincial CDC: Min Yu, Ruying Hu, Hao Wang. Tongxiang CDC: Yijian Qian, Chunmei Wang, Kaixu Xie, Lingli Chen, Yidan Zhang, Dongxia Pan, Qijun Gu. **Hunan** Provincial CDC: Yuelong Huang, Biyun Chen, Li Yin, Huilin Liu, Zhongxi Fu, Qiaohua Xu. Liuyang CDC: Xin Xu, Hao Zhang, Huajun Long, Xianzhi Li, Libo Zhang, Zhe Qiu.

## Appendix A Supplementary data

mmc1

mmc2

mmc3
