# Supplementary material for: Characteristics and correlates of sleep duration, daytime napping, snoring and insomnia symptoms among 0.5 million Chinese men and women
Source: Sleep Med. 2018 Apr;44:67–75. doi: 10.1016/j.sleep.2017.11.1131 (PMC5869948; doi:10.1016/j.sleep.2017.11.1131)
Supplement: mmc1 [file mmc1.docx]

| **Web Table 1: Mean sleep duration and proportion of reporting insomnia symptoms by selected characteristics, excluding individuals with prior disease, Major Depressive Episode and Generalised Anxiety Disorder.** | | | | | | |
| --- | --- | --- | --- | --- | --- | --- |
|  |  | **N** | **Mean sleep duration (hours)** | **Insomnia symptoms (%)** | **Short sleep (<= 6 hours) (%)** | **Long sleep (>=9 hours) (%)** |
| **All** |  | 355098 | 7.46 | 14.5 | 20.8 | 16.4 |
| **Age** | |  |  |  |  |  |
|  | 30-39 | 66448 | 7.76 | 9.9 | 12.1 | 20.2 |
|  | 40-49 | 119297 | 7.55 | 12.4 | 17.7 | 16.5 |
|  | 50-59 | 103840 | 7.34 | 16.5 | 23.8 | 14.7 |
|  | 60-69 | 49147 | 7.18 | 19.4 | 29.5 | 14.5 |
|  | 70-79 | 16366 | 7.03 | 22.1 | 35.8 | 15.2 |
| **Gender** | |  |  |  |  |  |
|  | Men | 144984 | 7.47 | 11.9 | 20.1 | 15.7 |
|  | Women | 210114 | 7.44 | 16.4 | 21.4 | 16.8 |
| **Region** | |  |  |  |  |  |
|  | Rural | 208922 | 7.58 | 16.0 | 19.0 | 20.7 |
|  | Urban | 146176 | 7.27 | 12.2 | 23.4 | 10.3 |
| **Highest education** | |  |  |  |  |  |
|  | No formal school / Primary school | 175661 | 7.45 | 15.4 | 22.3 | 17.8 |
|  | Middle school / High school | 160274 | 7.48 | 13.5 | 19.7 | 15.9 |
|  | Technical school / College / University | 19163 | 7.30 | 10.6 | 14.3 | 13.8 |
| **Annual household income (yuan)** | |  |  |  |  |  |
|  | < 10 000 | 101208 | 7.45 | 17.2 | 22.9 | 19.0 |
|  | 10 000-19 999 | 104624 | 7.49 | 14.4 | 20.6 | 17.6 |
|  | 20 000-34 999 | 87821 | 7.45 | 13.7 | 20.4 | 15.4 |
|  | >= 35 000 | 61445 | 7.47 | 13.6 | 19.2 | 15.7 |
| **Marital status** | |  |  |  |  |  |
|  | Married | 325522 | 7.47 | 14.2 | 20.3 | 16.5 |
|  | Widowed | 21148 | 7.21 | 20.9 | 28.3 | 13.6 |
|  | Separated / divorced | 5540 | 7.29 | 16.6 | 25.2 | 15.8 |
|  | Never married | 2888 | 6.89 | 13.2 | 21.7 | 15.3 |
| **Living alone** | |  |  |  |  |  |
|  | No | 346805 | 7.46 | 14.4 | 20.6 | 16.4 |
|  | Yes | 8293 | 6.93 | 19.2 | 29.6 | 14.3 |
| **Occupation** | |  |  |  |  |  |
|  | Manual worker | 218206 | 7.41 | 14.7 | 20.4 | 15.7 |
|  | Not in employment | 73354 | 7.44 | 16.0 | 21.1 | 18.8 |
|  | Office worker | 48140 | 7.41 | 12.9 | 19.6 | 14.0 |
|  | Other or not stated | 5606 | 7.26 | 13.8 | 20.3 | 19.5 |
|  | Unemployed | 9792 | 7.14 | 16.9 | 20.6 | 16.4 |
| **Smoking status** | |  |  |  |  |  |
|  | Never regular smoker | 241115 | 7.45 | 14.6 | 20.8 | 16.2 |
|  | Ex regular smoker | 15802 | 6.52 | 17.6 | 28.4 | 14.8 |
|  | Regular smoker | 98181 | 7.46 | 18.8 | 22.3 | 19.3 |
| **Alcohol** | |  |  |  |  |  |
|  | Never regular drinker | 157067 | 7.49 | 14.2 | 20.5 | 17.5 |
|  | Ex regular drinker | 3623 | 6.78 | 19.6 | 21.7 | 13.7 |
|  | Occasional drinker | 138590 | 7.46 | 14.6 | 20.5 | 16.4 |
|  | Weekly drinker | 55818 | 7.43 | 16.6 | 21.6 | 16.4 |
| **Physical activity (MET hours/day)**^1^ | |  |  |  |  |  |
|  | < 10 | 70766 | 7.52 | 15.1 | 21.3 | 20.4 |
|  | 10-29 | 184297 | 7.47 | 14.3 | 20.3 | 16.4 |
|  | >= 30 | 100035 | 7.36 | 14.3 | 22.3 | 13.9 |
| **BMI (kg/m²)^2^** | |  |  |  |  |  |
|  | <18.5 | 15467 | 7.34 | 19.1 | 24.4 | 15.6 |
|  | 18.5-24.99 | 232871 | 7.45 | 15.1 | 21.0 | 16.3 |
|  | 25-29.99 | 94984 | 7.50 | 12.6 | 19.7 | 17.0 |
|  | >= 30 | 11774 | 7.48 | 11.7 | 20.8 | 17.5 |
| **Waist Circumference (mm)** | |  |  |  |  |  |
|  | 200-699 | 53787 | 7.39 | 17.4 | 22.6 | 15.8 |
|  | 700-799 | 138818 | 7.45 | 15.1 | 21.1 | 16.3 |
|  | 800-899 | 111982 | 7.47 | 13.3 | 20.1 | 16.4 |
|  | 900-1499 | 50511 | 7.50 | 12.1 | 20.1 | 17.2 |
| **SBP (mmHg)^3^** | |  |  |  |  |  |
|  | <110 | 55193 | 7.42 | 15.4 | 21.3 | 15.7 |
|  | [110,120) | 71390 | 7.44 | 14.6 | 21.0 | 16.2 |
|  | [120,130) | 84684 | 7.45 | 14.4 | 20.9 | 16.2 |
|  | [130,140) | 61246 | 7.47 | 14.3 | 20.6 | 16.6 |
|  | >=140 | 82585 | 7.48 | 14.1 | 20.4 | 17.2 |
| *Adjusted for age, region and sex (where appropriate).* | | |  |  |  |  |

^1^ MET: Metabolic Equivalent Task; ^2^ BMI: Body Mass Index; ^3^ Systolic Blood Pressure
