# Supplementary material for: Characteristics and correlates of sleep duration, daytime napping, snoring and insomnia symptoms among 0.5 million Chinese men and women
Source: Sleep Med. 2018 Apr;44:67–75. doi: 10.1016/j.sleep.2017.11.1131 (PMC5869948; doi:10.1016/j.sleep.2017.11.1131)

Web Figure 1: Adjusted ORs for insomnia symptoms in individuals with (A) short sleep duration and (B) long sleep duration by mental health conditions and physical illness

	Sleep <= 6 hours: N	N insomnia symptoms	N no insomnia symptoms	OR (95% CI)	Sleep >= 9 hours: N	N insomnia symptoms	N no insomnia symptoms	OR (95% CI)
**Life satisfaction**								
Very satisfied / Satisfied	75790	32740	43050	1.00 (0.98, 1.02)	59086	3354	55732	1.00 (0.96, 1.04)
Neither satisfied nor dissatisfied	35674	17081	18593	1.27 (1.24, 1.29)	19742	1395	18347	1.19 (1.12, 1.26)
Unsatisfied / Very unsatisfied	6986	3784	3202	1.98 (1.89, 2.08)	2897	391	2506	2.78 (2.49, 3.11)
**Stressful life events**								
No	105505	46361	59144	1.00 (1.00, 1.00)	75177	4406	70771	1.00 (1.00, 1.00)
Yes	12945	7244	5701	1.67 (1.60, 1.73)	6548	734	5814	2.22 (2.04, 2.42)
**Self-rated health status**								
Excellent	17313	5338	11975	1.00 (0.97, 1.03)	13927	439	13488	1.00 (0.91, 1.10)
Good	26414	10424	15990	1.32 (1.29, 1.36)	25091	1409	23682	1.37 (1.30, 1.45)
Fair	55897	25918	29979	1.77 (1.74, 1.80)	33565	2186	31379	1.84 (1.76, 1.93)
Poor	18826	11925	6901	3.31 (3.21, 3.41)	9142	1106	8036	3.74 (3.50, 4.00)
**Major depressive episode (MDE)**								
No	116925	52377	64548	1.00 (1.00, 1.00)	81232	4977	76255	1.00 (1.00, 1.00)
Yes	1525	1228	297	4.81 (4.22, 5.47)	493	163	330	6.91 (5.67, 8.43)
**Generalized anxiety disorder (GAD)**								
No	117844	53101	64743	1.00 (1.00, 1.00)	81563	5080	76483	1.00 (1.00, 1.00)
Yes	606	504	102	5.46 (4.40, 6.78)	162	60	102	9.06 (6.51, 12.6)
**Any prior disease**								
No	74807	31659	43148	1.00 (1.00, 1.00)	58694	3289	55405	1.00 (1.00, 1.00)
Yes	43643	21946	21697	1.41 (1.37, 1.45)	23031	1851	21180	1.48 (1.39, 1.58)
**Coronary heart disease**								
No	112906	50618	62288	1.00 (1.00, 1.00)	79838	4987	74851	1.00 (1.00, 1.00)
Yes	5544	2987	2557	1.60 (1.51, 1.69)	1887	153	1734	1.61 (1.35, 1.92)
**Stroke or TIA**								
No	115874	52347	63527	1.00 (1.00, 1.00)	79981	5027	74954	1.00 (1.00, 1.00)
Yes	2576	1258	1318	1.26 (1.16, 1.37)	1744	113	1631	1.08 (0.88, 1.32)
**Emphysema/bronchitis**								
No	114060	51105	62955	1.00 (1.00, 1.00)	79788	4966	74822	1.00 (1.00, 1.00)
Yes	4390	2500	1890	1.46 (1.38, 1.56)	1937	174	1763	1.45 (1.23, 1.71)
**Cancer**								
No	117667	53199	64468	1.00 (1.00, 1.00)	81284	5112	76172	1.00 (1.00, 1.00)
Yes	783	406	377	1.28 (1.11, 1.48)	441	28	413	0.92 (0.63, 1.36)
**Diabetes**								
No	109761	49612	60149	1.00 (1.00, 1.00)	77216	4828	72388	1.00 (1.00, 1.00)
Yes	8689	3993	4696	1.09 (1.04, 1.14)	4509	312	4197	1.08 (0.96, 1.22)

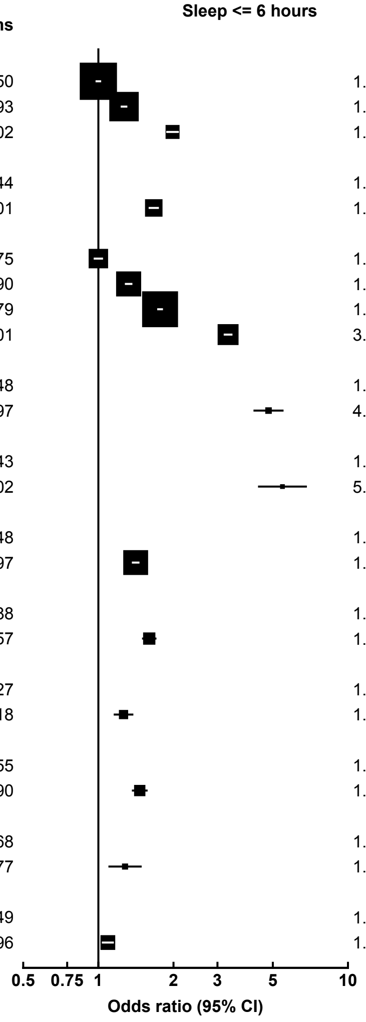

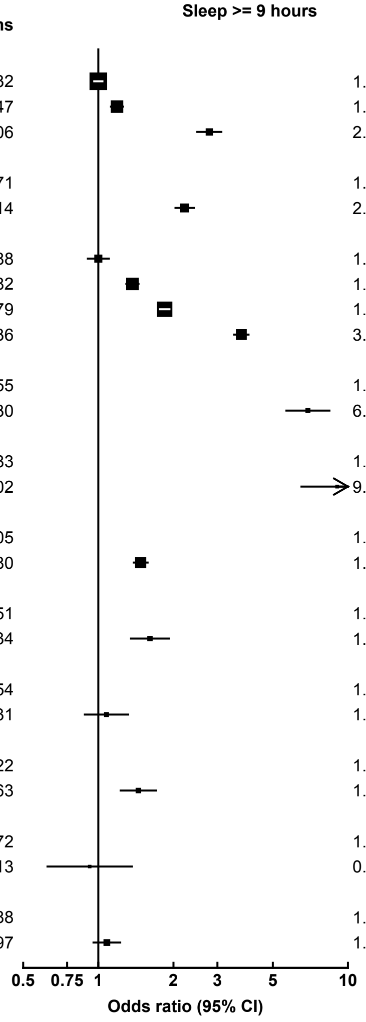

Supplement: mmc2 [file mmc2.pdf]
