# Supplementary figures and images for: Characteristics and correlates of sleep duration, daytime napping, snoring and insomnia symptoms among 0.5 million Chinese men and women
Source: Sleep Med. 2018 Apr;44:67–75. doi: 10.1016/j.sleep.2017.11.1131 (PMC5869948; doi:10.1016/j.sleep.2017.11.1131)

**Web figure 1: Proportion with insomnia symptoms by sleep duration adjusted for region**

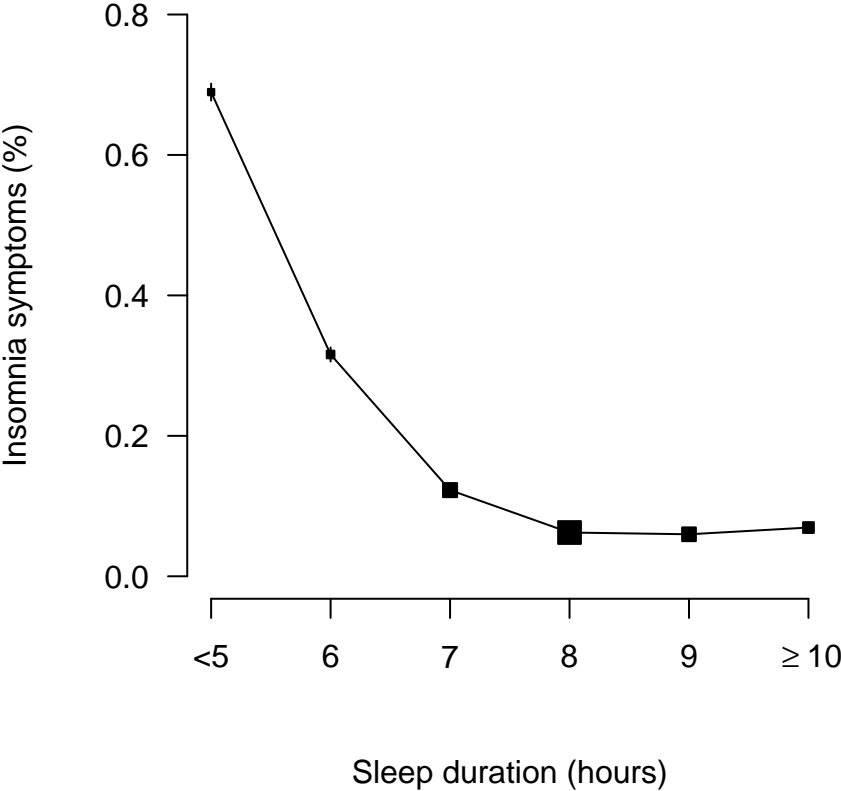

Supplement: mmc3 [file mmc3.pdf]
